# Anatomical Variation Quality Assessment Tool (AVQAT): Structured Guide for Reporting and Assessing the Quality of Anatomical Case Reports

**DOI:** 10.1002/ca.70141

**Published:** 2026-05-01

**Authors:** Vojtěch Kunc, David Kachlík, Michal Beneš

**Affiliations:** ^1^ Department of Anatomy, Second Faculty of Medicine Charles University Prague Czech Republic; ^2^ Center of Endoscopic, Surgical and Clinical Anatomy (CESKA) Second Faculty of Medicine, Charles University Prague Czech Republic; ^3^ 1st Department of Orthopaedics, First Faculty of Medicine Charles University and Motol University Hospital Prague Czech Republic; ^4^ Clinic of Trauma Surgery Masaryk Hospital Usti nad Labem Czech Republic

**Keywords:** anatomical variability, case report, quality assessment

## Abstract

Anatomical variations are common and often clinically important, yet their description frequently relies on isolated case reports or small series with heterogeneous reporting quality. Incomplete documentation of specimen characteristics, inconsistent anatomical definitions and terminology, insufficient description of visualization methods, limited quantitative measurements, and suboptimal figures all reduce reproducibility and limit comparison and data synthesis. To address the lack of an anatomy‐specific quality assessment, the Anatomical Variation Quality Assessment Tool (AVQAT) is introduced as a structured instrument intended for authors, reviewers, and editors. AVQAT comprises two sections organized into nine domains. Section A evaluates technical and descriptive quality across six domains: general aspects, specimen characteristics, anatomical definition, methods of visualization, quantitative description and measurements, and quality and use of figures. Section B evaluates interpretative and contextual quality in three domains: differential diagnostic considerations, identification and integration of previous studies, and ethical and regulatory considerations. Each domain contains targeted items that can be assessed systematically using “Yes,” “No,” or “Not applicable” responses. The tool is designed to complement, not replace, generic case‐report guidance by focusing on the specific demands of anatomical documentation and thereby facilitating higher‐quality reporting and more reliable evidence synthesis in scoping and systematic reviews.

## 1 Introduction

Anatomical variations are common and frequently clinically relevant features of human morphology. Their systemic description deepens our understanding of human anatomy, leading to safer surgical practice, more accurate imaging interpretation, reduced perioperative complications, and enabling the use of variants in surgical procedures (such as tendon and nerve transfers, flap design, etc.) (Alraddadi 2021). For many structures, however, current knowledge is largely derived from isolated case reports or small series that describe unexpected findings in body donors, in imaging studies, or during surgical procedures. These reports are indeed valuable, and the number of published cases increases every year (Wysiadecki et al. 2024). Their scientific value depends heavily on the clarity, completeness, and methodological rigor, including metric data with which variations are documented. Inconsistent reporting, whether in anatomical definitions, baseline characteristics, imaging modalities, dissection methods, measurements, or figures, limits reproducibility and meaningful comparisons between studies.

Anatomical variations should be regarded not merely as incidental morphological findings, but as potentially important determinants of clinical decision‐making, surgical safety, and anatomical education. Their recognition may influence operative planning, interpretation of radiological findings, avoidance of iatrogenic injury, and selection of reconstructive options (Riley 2013).

Despite the importance of reporting anatomical variations, there is no widely accepted, anatomy‐specific quality assessment tool to guide authors, reviewers, and editors in assessing the quality of anatomical variation case reports, as the existing tools for case reports do not address the specific requirements of anatomical documentation (Gagnier et al. 2013; Munn et al. 2020). Case reports may omit critical information, making it difficult to assess the rare variants through different methods of data synthesis to gain stronger evidence.

To address this gap, we developed a structured quality assessment tool specifically tailored to case reports of anatomical variants.

## 2 Anatomical Variation Quality Assessment Tool (AVQAT)

The structure of AVQAT was derived from general reporting guidance for case reports and methodological literature on appraisal tools, but its final content was specifically adapted to the context of anatomical variation research. Particular emphasis was placed on elements that are essential for anatomical reproducibility and interpretation, including precise anatomical terminology, specimen characteristics, methods of visualization, quantitative measurements, figure quality, differential diagnostic considerations, integration of previous literature, and ethical and regulatory aspects.

The AVQAT is organized into two sections with nine domains. Section A focuses on the technical and descriptive quality of the report, covering six domains: (1) General aspects; (2) Specimen characteristics; (3) Anatomical definition; (4) Methods of visualization; (5) Quantitative description and measurements; and (6) Quality and use of figures. Section B addresses interpretative and contextual aspects in three domains: (1) Differential diagnostic considerations; (2) Identification and integration of previous studies; and (3) Ethical and regulatory considerations. Each domain consists of specific questions that can be answered “Yes,” “No,” or “Not applicable” systematically by authors and reviewers.

### 2.1 Section A

#### 2.1.1 Domain 1: General Aspects

- Is the title self‐explanatory and followed by the specifying words “case report”?
- Are two‐to‐six keywords relevant to the contents of the case report provided?
- Is either structured or non‐structured abstract summarizing the key findings provided?
- Is the text structured to provide (a) a brief rationale and uniqueness of the case, (b) an independent section describing the case, and (c) discussion of relevant scientific literature with clear conclusions?

#### 2.1.2 Domain 2: Specimen Characteristics

- Are the type and origin of the specimen clearly specified (e.g., body donor, living patient, and volunteer)?
- Is the relevant population background of the subject reported?
- Are the key subject characteristics provided (e.g., age, sex, side, and anatomical region)?
- Is it an accidental finding, or was there any direct or indirect hint/symptom reported before?

#### 2.1.3 Domain 3: Anatomical Definition

- Is it clearly presented how many anatomical variations are described in the case and what their relationship is (e.g., one or more, and if more, related or non‐related)?
- Is the clear and detailed anatomical definition of the variation provided?
- In case of more anatomical variations reported, are all concomitant variations explicitly described and defined?
- Is the normal or reference anatomy clearly described and referenced for comparison?
- Is the standardized anatomical terminology used consistently throughout the text?
- Are the definitions used consistently in the text and in agreement with the figures and legends?

#### 2.1.4 Domain 4: Methods of Visualization

- Are the primary methods used to identify and document the variation explicitly stated (e.g., dissection, imaging, perioperative findings, histology, inspection, and manual examination)?
- Are any secondary or confirmatory methods listed where applicable?
- Is it stated whether the contralateral side or additional specimens were examined for comparison?
- Are the technical details sufficiently described?
  - Dissection:
    - Is the fixation method and condition of the donor body described?
    - Is the context of the finding stated (e.g., incidental finding during a teaching course vs. targeted search)?
    - Is the dissection approach described in sufficient detail for potential reproducibility?
  - Imaging:
    - Is the imaging modality and key parameters/protocol described (e.g., slice thickness, sequences, segmentation, reconstruction, frequency of probe, and usage of contrast)?
    - Is the software or workstation used for visualization and measurements specified?
  - Perioperative findings:
    - Is the surgical procedure during which the finding was made described?
    - Is the surgical approach specified?
    - Is the patient's position during the procedure specified?
    - Are perioperative adjuncts described if used (e.g., C‐arm, tourniquet, microscope, and magnification)?
  - Histology:
    - Are tissue processing methods (fixation and embedding) and staining protocols described?
    - Are magnification and microscopic techniques specified?
    - Are the histological features relevant to the anatomical diagnosis clearly reported?
  - Manual examination/Aspection:
    - Is the method of examination specified and described in detail?
    - Is the examination performed once by one or more researchers, or repeatedly by one or more researchers?

#### 2.1.5 Domain 5: Quantitative Description & Measurements

- Are all measurements clearly defined (including reference points and planes) with sufficient precision?
- Are relevant dimensions reported (e.g., length, width, thickness, diameter (specifying if internal or external), distances, and angles)?
- Is the methodology of measurement clearly described (including instruments and software used)?
- Are appropriate units and numerical precision used consistently?
- Are potential sources of measurement bias or error identified and clearly stated?

#### 2.1.6 Domain 6: Quality and Use of Figures

- Is the key anatomical finding clearly documented in at least one appropriate figure (photograph, imaging slice, 3D reconstruction, video, etc.)?
- If appropriate, are the figures supported by an illustrative and self‐explanatory scheme?
- Are the figures presented in sufficient resolution and clarity to identify the relevant structures?
- In case of dissection image, are the figures presenting a well processed dissection with cleaned region of interest and devoid of disturbing objects (e.g., fingers in gloves, not necessary instruments, surrounding non‐related objects) with unified background?
- Are the figures clearly labeled, with consistent terminology matching the text?
- Are figure legends sufficiently detailed (modality, side, orientation, and key structures) to allow understanding without extensive reference to the main text?
- Are orientation markers (directional arrows) and/or scale bars provided?

### 2.2 Section B

#### 2.2.1 Domain 7: Differential Diagnostic Considerations

- Are the main differential diagnostic possibilities explicitly mentioned?
- Is the distinction between the final interpretation and alternative possibilities clearly explained, justified, and referenced?
- Are clinical, imaging, or histological correlations available to support the final interpretation?

#### 2.2.2 Domain 8: Identification and Integration of Previous Studies

- Is the rationale behind the rarity or uniqueness of the case report described?
- Are relevant previous studies (including scoping reviews, systematic reviews or meta‐analyses, if available), textbooks, and atlases identified, or a clear statement provided that none exists, including bias of language barrier?
- Is a clear comparison with previously reported variations or findings provided (similarities and differences)?
- Does the review of prior literature include historical and non‐indexed sources when relevant to reduce the risk of misclassifying a known variant as novel?
- Is the developmental background of the variation discussed, including embryological and, where relevant, phylogenetic/comparative anatomical perspectives?

#### 2.2.3 Domain 9: Ethical and Regulatory Considerations

- Is a statement on ethical approval explicitly provided, even if waived (e.g., institutional review board, ethics committee, and number of approval)?
- Is the anonymity and confidentiality of the subject(s) ensured both in the text and figures?
- Is the inclusion of donor(s)/patient(s) appropriately acknowledged?

## 3 Discussion

Over the past decade, anatomical research has experienced widespread tools that aimed to enhance the quality of original studies (Tomaszewski et al. 2017) or systematic reviews and meta‐analyses (D'Antoni et al. 2022; Henry et al. 2016; Yammine 2014). These tools were tempered from generally accepted guidelines in medical research, such as the PRISMA (Page et al. 2021) or STROBE (von Elm et al. 2007) guidelines, to reflect the needs of anatomical research. Nowadays, clinical case reports are encouraged to follow the CARE guidelines (Gagnier et al. 2013), which cover critical areas. However, advancements in anatomy research seek for a more detailed and specific tool. This article introduces, to our knowledge, the first anatomy‐specific quality assessment tool designed for case reports of anatomical variations. By structuring the quality assessment into two sections and nine domains that focus on both the technical description and anatomical context, the AVQAT aims to address shortcomings in case reports of anatomical variations. Common problems include incomplete specimen data, insufficiently defined variations, poor graphical presentation of the case, inappropriate and inconsistent terminology, and limited reporting of the methods used. Rather than replacing existing generic guidelines for case reports, it is intended as a complementary instrument that focuses on the demands of case reports on anatomical variations.

Anatomical case reports involving body donors should also adhere to current ethical recommendations on humanizing donor terminology, with potentially depersonalizing terms such as *cadaver* or *specimen* used cautiously and replaced by *donor*‐centered language where appropriate (Tabira et al. 2026).

A key strength of the AVQAT lies in the emphasis placed on Section A, which covers specimen characteristics, anatomical definition, methods of visualization, quantitative measurements, and the quality of figures. This section is important for all anatomical case reports, even for reports that focus purely on morphology without explicit clinical implications. Section B extends the scope of quality assessment beyond description to interpretation and context, while paying attention to frequently overlooked ethical aspects (Henry et al. 2018; Iwanaga et al. 2021; Iwanaga et al. 2022; Saglam et al. 2025). Differential diagnostic considerations help prevent misclassification of anatomical variations as pathological findings or vice versa.

The AVQAT was primarily developed to enable better synthesis of anatomical case report data through systematic and scoping reviews, which are often hindered by a lack of essential details on specimen characteristics (Omotoso et al. 2026), methods, and measurements. Moreover, the anatomical definition of reported structures as well as the proper anatomical terminology is often missing. Over time, consistent use of such a tool could contribute to a better understanding of rare anatomical variants.

The AVQAT has several other uses. For authors, it can serve as a structured checklist during study design, data collection, and manuscript preparation, helping ensure that key elements are not omitted. For reviewers and editors, it offers a transparent framework for evaluating the quality and completeness of case reports of anatomical variations, supporting more consistent and constructive feedback. For educators and trainees, the domains can be used as a teaching scaffold to illustrate what constitutes a high‐quality anatomical case report and to guide students in documenting their own findings during dissection courses or clinical rotations.

Several limitations of the tool should be acknowledged. Although the domains and items are based on recognized principles of anatomy research, the tool has not yet undergone formal validation, such as testing of inter‐rater and intra‐rater reliability. Moreover, some degree of subjectivity in scoring is unavoidable, particularly for items related to “sufficient” figure legends or figure quality. It must be noted that this tool is based on an expert opinion initiative, it should be regarded as expert opinion on the scale of evidence levels. Finally, it must be mentioned that this tool does not aim to be superior to the author guidelines of scientific journals but should be regarded as a tool to strengthen anatomical research. Presentation of rare anatomical variations at scientific meetings and discussion with expert anatomists and clinicians before finalizing the manuscript may provide important feedback that strengthens the interpretation, contextualization, and conclusions of the report.

## Funding

The authors have nothing to report.

## Ethics Statement

The work has been conducted in accordance with The Code of Ethics of the World Medical Association (Declaration of Helsinki) and its later amendments.

## Consent

The authors have nothing to report.

## Data Availability

Data sharing not applicable to this article as no datasets were generated or analysed during the current study.
